# What can AI do for mental health care

**DOI:** 10.1192/j.eurpsy.2025.239

**Published:** 2025-08-26

**Authors:** U. Volpe

**Affiliations:** Deaprtment of Clinical Neurosciences/DIMSC, Università Politecnica delle Marche, Ancona, Italy

## Abstract

**Abstract:**

Artificial intelligence (AI) is reshaping mental health care by improving diagnosis, treatment, and patient support. AI-driven tools streamline administrative tasks, enhance clinical documentation, contribute to medical education, and enable continuous symptom monitoring. Additionally, AI augments text-based support programs, offering accessible psychological interventions. However, while AI applications have been assessed for perceived empathy, their impact on clinical outcomes remains uncertain, highlighting the need for evidence-based implementation.

AI can immediately enhance efficiency in routine clinical tasks. Automation optimizes billing processes and reduces clerical burdens, allowing clinicians to focus more on patient care. In clinical documentation, AI-powered transcription and natural language processing (NLP) help generate structured medical records. AI also supports medical education by offering adaptive learning, personalized training, and real-time feedback through large-scale data analysis.

Beyond administrative support, AI plays a role in patient monitoring and early intervention. AI algorithms analyze speech, facial expressions, and behavioral data from smartphones and wearables, detecting mood fluctuations and early psychiatric symptoms. This real-time analysis can facilitate timely interventions and improve overall mental health care. Additionally, AI-powered chatbots and virtual therapists are increasingly used in digital mental health services, providing immediate, text-based psychological support. However, rigorous studies are needed to assess their effectiveness in improving clinical outcomes. A well-established framework for technology evaluation in mental health highlights five key areas for development: equity, privacy, evidence, clinical engagement, and interoperability. Addressing these factors is crucial to ensuring AI-driven solutions are accessible, secure, scientifically validated, clinically integrated, and capable of working across diverse health systems. By prioritizing these advancements, AI can move from theoretical promise to practical application, meaningfully improving mental health care delivery.

**Disclosure of Interest:**

None Declared

